# Microstructure Evolution Behaviors and Mechanical Properties of 6082-0.2Zr-0.3Er Alloy During the Pre-Hardened Hot Forming Process

**DOI:** 10.3390/ma18030616

**Published:** 2025-01-29

**Authors:** Feng Huang, Daoling Zhang, Yingchuan Deng, Zhe Cheng, Qian Sun, Zhili Hu, Lin Hua

**Affiliations:** 1 Hubei Longzhong Laboratory, Xiangyang 441022, China; 2 Hubei Key Laboratory of Advanced Technology of Automotive Components, Wuhan University of Technology, Wuhan 430070, China

**Keywords:** 6082-0.2Zr-0.3Er alloy, deformation and heat treatment process, microstructure, mechanical properties

## Abstract

Combining a comparative experimental study and theoretical analysis, the feasibility and corresponding action mechanisms of the pre-hardened hot forming (PHF) process applied to 6082-0.2Zr-0.3Er alloy were discussed. The results indicate that unlike a traditional process, after PHF treatment, both the grains and the precipitates at the grain boundaries show a certain directional arrangement, and the texture coefficients of (220) and (200) are enhanced. Although the average size of the second phase is almost the same, the proportion of the fine second phase in the PHF-treated sample is larger. In addition to shortening the processing time and improving the production efficiency, the PHF process was proven to be beneficial for the improvement in the comprehensive mechanical properties of this 6082-0.2Zr-0.3Er alloy, with the product of strength and elongation increased by about 12.1% under the given conditions. Furthermore, with the optimization of process parameters, the improvement effect is expected to be further enhanced.

## 1. Introduction

At present, with the development of automotive lightweighting, aluminum alloy has become an important substitute for steel [1,2]. The process of cold forming after aging reinforcement often faces issues such as long aging times, poor room-temperature formability, and spring back after forming [3,4,5], which limits its application in industrial fields [6,7,8]. On the other hand, the hot forming followed by the heat treatment process (such as T6) often suffers from long process flows, high energy consumption, and deformation in the subsequent heat treatment, which requires further correction [9,10,11]. Over the years, significant efforts have been made to address various issues in aluminum alloy forming, such as increasing forming temperature [12] and changing deformation mode [13]. A hot stamping technology for aluminum alloys, known as hot forming and cold-die quenching (HFQ^®^), was patented by Lin et al. [14]. In this process, aluminum alloy sheets are solution-treated and then transferred to a pair of water-cooled dies for stamping and in-die quenching, followed by low-temperature short-time aging. Using this HFQ^®^ forming process, Ford has successfully trial-produced 7075-aluminum alloy B-pillars. Although formability can be significantly improved through this forming method, the solution heat treatment and the subsequent artificial aging result in high energy consumption and low production efficiency [15,16]. As a result, researchers have focused on improving the HFQ^®^ process, such as finding ways to enhance the efficiency of solution treatment [17] and aging treatment [6,7]. However, studies [18,19,20,21] have shown that faster solution and aging heat treatments are not ideal for shortening the HFQ^®^ production cycle time while ensuring quality.

Hua et al. [22] proposed a novel forming technology called pre-hardened hot forming (PHF). In this technology, the raw sheet is rapidly heated to below the solution temperature for pre-aging and then quickly transferred to a cold die in the pre-aging strengthened state (Tx) for stamping. During stamping, a component can be formed by applying a certain pressure and holding the pressure for a period of time, and no subsequent aging treatment is required after the component is formed. It is clear that this method not only lowers the heating temperature and reduces the heating time but also eliminates the need for subsequent aging treatment after forming, thus significantly shortening the production cycle and making it more suitable for the mass production of parts. Currently, many studies have verified the feasibility of this PHF process and found that its action mechanism is closely related to the precipitation behaviors of related phases in the treatment process [23,24,25]. It should be noted that almost all the previous studies were carried out using 7xxx aluminum alloy, that is, Al-Zn-Mg-Cu alloy, while research on other aluminum alloys was rare. Therefore, the applicability of this PHF process on other aluminum alloys and the relevant action mechanisms need to be further verified and revealed.

In addition to 7xxx aluminum alloy, 6xxx aluminum alloy, such as 6082, is another commonly used aluminum alloy whose strength is lower than that of 7xxx aluminum alloy. The previous experimental study of our research group indicated that the mechanical properties of 6082 aluminum alloy can be effectively improved by Zr-Er composite microalloying, and 6082-0.2wt.%Zr-0.3wt.%Er (hereinafter referred to as 6082-0.2Zr-0.3Er) exhibited the best overall performance. Based on this, selecting 6082-0.2Zr-0.3Er as the research object, the microstructures and mechanical properties of the alloy treated with PHF were characterized and measured and combined with the comparative analysis with the corresponding traditional process, the feasibility and corresponding action mechanisms of the process applied to this aluminum alloy were discussed.

## 2. Experimental Materials and Methods

**Figure 1:**
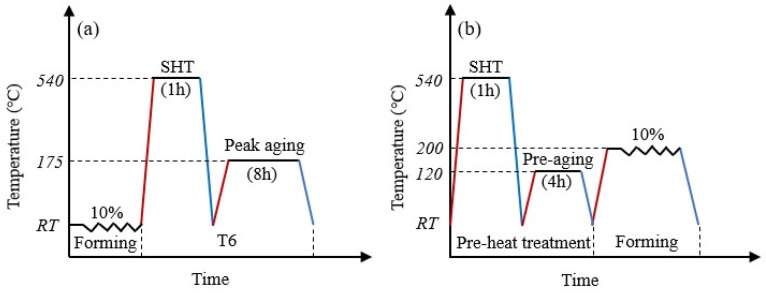
Flow chart of the processes used in this paper: (**a**) traditional (**b**) PHF.

The as-cast 6082-0.2Zr-0.3Er alloy prepared by our research group by melting in the miniature furnace was used as raw materials for all the deformation and heat treatment experiments. The detailed casting process is as follows: The Al block was melted and held at 780 °C for 30 min. Subsequently, Zn, Mg, and other intermediate alloys were melted at 740 °C and held for 15 min before being poured into a graphite mold at room temperature. Table 1 lists the chemical composition of the alloy. As shown in Figure 1, two different deformation and heat treatment processes were used in this paper to treat the cast ingot. In the traditional process, the ingot was first cold compressed by 10% and then subjected to T6 (solution followed by artificial peak aging) heat treatment. For the PHF process, the ingot was first treated with a solid solution and pre-aging and finally subjected to hot compression of 10% at a temperature slightly higher than that of aging. Here, the compression ratio of cold compression in the traditional process and hot compression in the PHF process was the same, both of which were used to simulate the component forming process in actual production. The related processing parameters based on the preliminary research outcomes of our previous research [10,22,24,25] are listed in Table 2.

Rectangular blocks with dimensions of 10 × 10 × 5 mm were cut from each treated sample for structural characterization. After grinding, polishing, and etching with a Keller’s reagent (HF:HCl:HNO_3_ = 2:3:5), the metallographic structures of each sample were characterized by an optical microscope (OM). An Axio Scope A1 optical microscope was used for OM observations. The microstructures were observed by scanning electron microscopy (SEM) in the backscattered electron (BSE) mode, and the chemical composition of the phases present in the microstructure was identified using energy dispersive X-ray spectroscopy (EDS). The SEM observations were carried out using a JSM-iT800 field emission scanning electron microscope, and the chemical composition analysis was performed using an energy spectrum analyzer fitted to the microscope. The physical phase composition of each sample was analyzed using XRD at a scan rate of 5°/min and a scan range of 20°–90°. XRD tests were performed on an Empyrean-type X-ray diffractometer at 40 kV of voltage and 30 mA of current with Cu Kα. It is worth noting that for each treated sample, OM, SEM, and XRD characterization were performed on the same block, with the only difference being that the OM characterization sample was etched while the other two were only polished.

**Figure 2:**
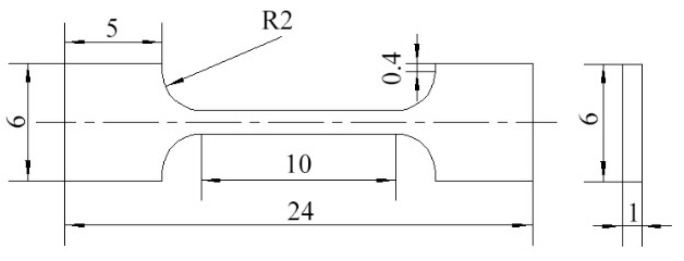
Schematic diagram of the tensile specimen size (unit: mm).

Using the sample shown in Figure 2, the tensile properties were tested at room temperature using a UTM4503 electronic universal testing machine with a test speed of 0.5 mm/min. In order to improve the accuracy and reliability of the testing data, five specimens were cut from each sample for testing. The five sets of experimental data were averaged and then the curve closest to the average was selected for plotting. After the tests, the corresponding fracture morphologies were observed using SEM in secondary electron (SE) mode.

## 3. Results and Discussion

### 3.1. Microstructure Characteristics

**Figure 3:**
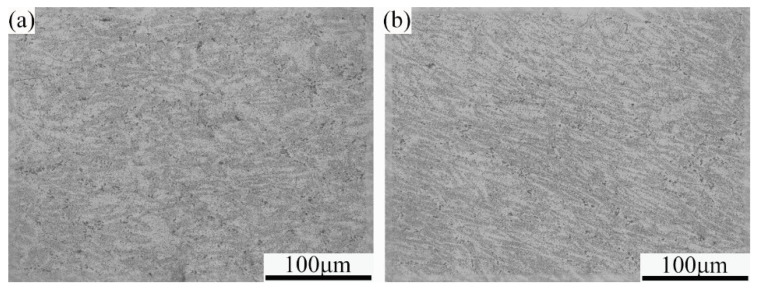
OM structures of the samples treated with different deformation and heat treatment processes. (**a**) Traditional; (**b**) PHF.

The typical metallographic structures of the samples treated with different processes are shown in Figure 3. As can be seen from the figure, the sample treated with the traditional process exhibits a dendritic structure with relatively coarse grains, and the grain orientation is not clearly defined. In contrast, in the PHF-treated sample, the grain size is smaller, and its aspect ratio is clearly increased, showing a certain degree of grain orientation. The above results indicate that the deformed structure formed during hot compression at 200 °C after pre-aging is retained to a certain extent under the PHF process, which is undoubtedly conducive to the coupling strengthening between the pre-aging strengthening and the subsequent deformation strengthening.

**Figure 4:**
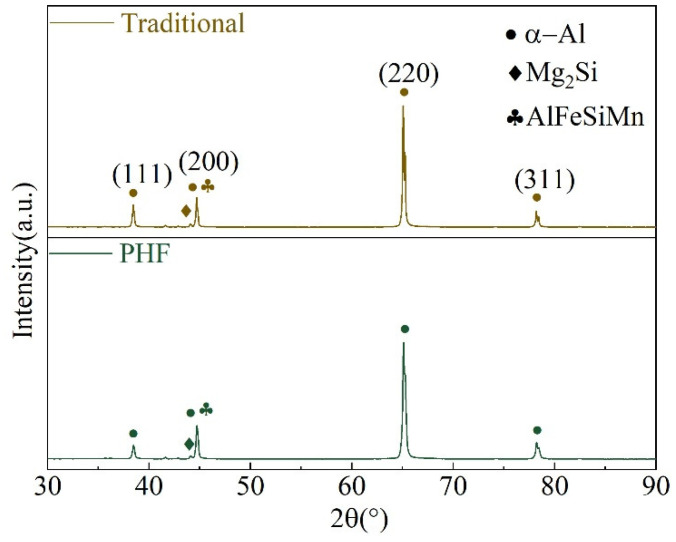
XRD patterns of the sample treated with different deformation and heat treatment processes.

Figure 4 shows the XRD patterns of the samples treated with a different process. As illustrated, both the samples are mainly composed of (111), (200), (220), and (311) oriented α-Al matrix and Al(Fe,Mn)Si phase, with the (220) oriented α-Al matrix being the most prominent. Attributing to the lower temperature and shorter time of pre-aging in the PHF process, the corresponding precipitates were not completely transformed into Mg_2_Si. Thus, the diffraction peak of the Mg_2_Si phase in the XRD pattern of the PHF-treated sample is slightly reduced. In addition, comparing the measured diffraction angles of different orientations with the corresponding standard cards, it is not difficult to find that each diffraction angle has a slight deviation, which is caused by lattice distortion. The lattice parameter a of the sample matrix was calculated according to the XRD results. The calculated results indicate that the corresponding lattice parameters of the sample treated with traditional process and PHF are 4.0527 Å and 4.0563 Å, respectively. It is clear that the peak aging is not reached during pre-aging; that is, the aging precipitate is not complete, and the solid solution elements in the aluminum matrix are more, thus leading to the lattice distortion of the PHF-treated sample being larger than that treated with the traditional process.

Comparing the diffraction peaks of the α-Al matrix in the two samples, it is clear the crystal texture of this 6082-0.2Zr-0.3Er alloy has changed under different treatment processes. The calculation results of the corresponding crystallographic texture coefficients are shown in Table 3. As illustrated, the matrix crystal texture of the sample treated with the traditional process in descending order is (220) → (311) → (200) → (111), with the corresponding texture coefficients are 72.5%, 13.6%, 9.2%, and 4.7%, respectively. For PHF treated sample, the (220) orientation remains dominant, but the corresponding matrix crystal texture in descending order changes into (220) → (200) → (311) → (111). Comparing the texture coefficients of the two samples, it is evident that the texture of (111) and (311) in the PHF-treated sample was weakened, with the corresponding texture coefficients decreased by 3.3% and 2.1%, respectively. On the other hand, the texture of (220) and (200) was enhanced, with the corresponding texture coefficients increased by 2.3% and 3.1%, respectively. Based on this, it can be inferred that the grains of Zr and Er micro alloyed 6082 aluminum alloy tend to grow along (220) and (200) crystal planes during dynamic recrystallization under the PHF process.

**Figure 5:**
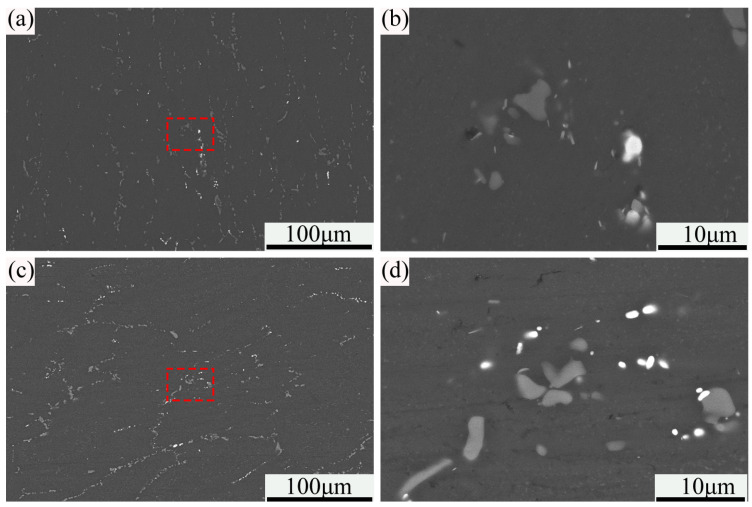
SEM microstructures of the samples treated with different processes: (**a**,**b**) traditional; (**c**,**d**) PHF.

Figure 5 shows the SEM microstructures of the samples treated with a different process. As illustrated, in the microstructure of the sample treated with the traditional process, the blocky gray phase is fine and evenly distributed. At the same time, numerous small and short rod-like blocky white phases were precipitated. For the sample treated with PHF, a large number of gray and white phases were also precipitated in the microstructure. The difference is that the gray phases in this sample are mainly distributed along the grain boundaries and show a certain directional orientation together with the matrix grains. In addition, compared with the sample treated by the traditional process, the white particle phase in the sample treated by PHF is finer and more evenly distributed.

**Figure 6:**
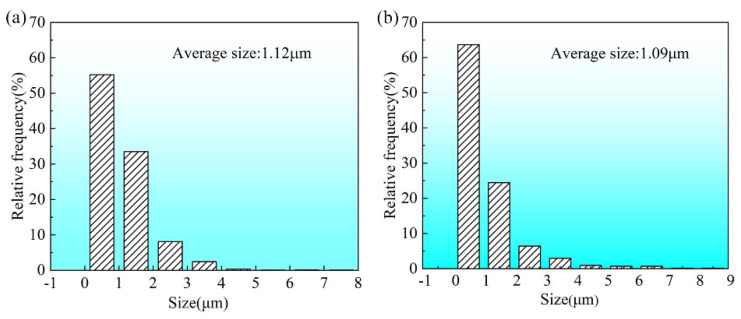
Size distribution of the second phase in the samples treated with different processes. (**a**) Traditional; (**b**) PHF.

In order to facilitate analysis, the other precipitated phases in the microstructure, except the matrix, are collectively referred to as the second phase. For further quantifying the size of the second phase in the two processes, Image J 2.0 software was used to statistically analyze the size distribution in multiple regions under the same magnification as Figure 5b,d, and the analyzing results are shown in Figure 6. As can be seen from the figure, the average size of the precipitated second phase in the sample treated with PHF is similar to that of the sample treated under the traditional process, which is 1.09 µm and 1.12 µm, respectively. However, comparing the size distribution of the second phase in the two samples, it is not difficult to find that the corresponding distribution is more concentrated in the sample treated by PHF, which is manifested by a higher proportion of small size. It is clear that the fine and evenly distributed second phase helps to improve the mechanical properties of the alloy, particularly the plasticity.

**Figure 7:**
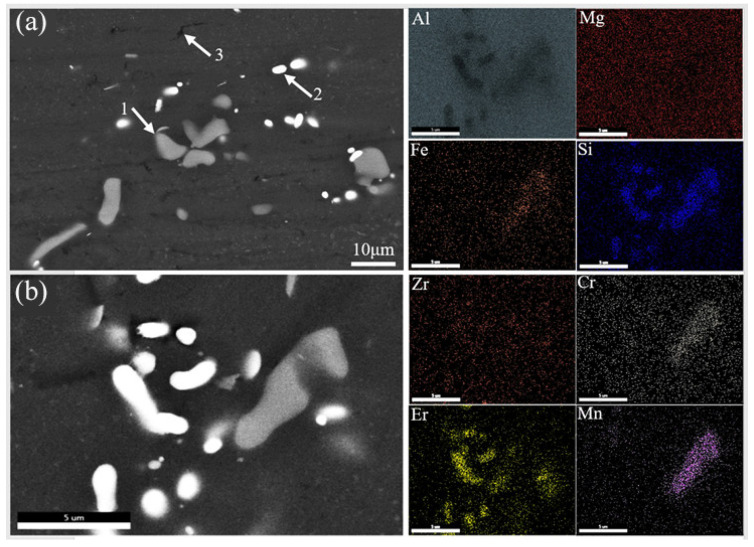
EDS analysis of the sample treated by PHF: (**a**) detecting points; (**b**) mapping area.

In order to further analyze the chemical composition of each phase and element distribution in the microstructure, EDS point and mapping analysis were carried out on the PHF-treated sample, and the corresponding detecting results are shown in Figure 7 and Table 4. The gray phase (point 1) is rich in Mn, Cr, and Fe in addition to Al, Si, and Mg, and the (Mn+Fe)/Si ratio is close to 1. Combined with the above XRD analyzing the result and corresponding reference [26], it is speculated that it should be the Al(MnFe)Si phase, with some Cr soluted. For the white phase (point 2), which is rich in Al, Si and Er, it is presumed to be the AlErSi phase. It should be noted that, due to the low content and small size of this phase, it was not detected in the above XRD analysis. For point 3, in addition to Al, it also contains Mg and Si. Combined with relevant studies [27,28], it is speculated that this fine black phase is Mg_2_Si. Because of the incomplete aging, the precipitated Mg_2_Si reinforcement phase in this PHF-treated sample is smaller. In addition, as can be seen from EDS mapping, Zr is evenly distributed in the alloy, which is clearly different from another microalloying element, Er.

### 3.2. Mechanical Properties

**Figure 8:**
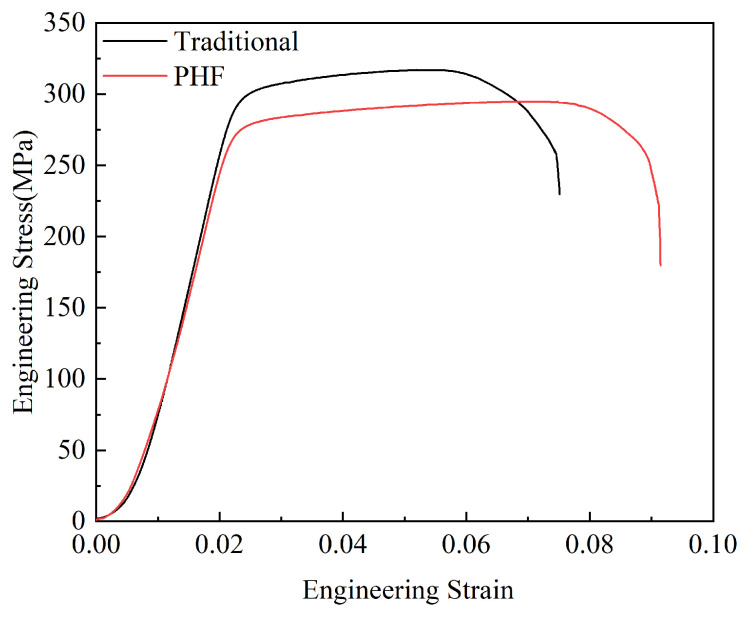
The engineering stress–strain curves of the two samples at room temperature.

The typical stress–strain curves of the two samples at room temperature are shown in Figure 8. In addition, the yield strength, ultimate tensile strength (UTS), and elongation obtained from the stress–strain curves are shown in Table 5. Compared with the traditional process, it is clear that the strength of the alloy decreased slightly, and the plasticity increased after being treated by PHF under the processing conditions used in this study. Specifically, the yield strength and tensile strength of the PHF-treated sample decreased from 309 MPa and 319 MPa under the traditional process to 280 MPa and 295 MPa, that is, with a decrease of 10.4% and 8.1%, respectively. On the contrary, the elongation increased from 7.5% to 9.1%, that is, with an increase of 21.3%.

The mechanisms determining the strength of the two samples are different. In the traditional process, strength is primarily governed by artificial aging, whereas in the PHF process, both deformation strengthening and aging strengthening contribute to the final strength. As the results show, for this 6082-0.2Zr-0.3Er aluminum alloy, the effect of aging strengthening on the alloy strength is greater than that of deformation strengthening under this experimental condition. The decrease in tensile strength and increase in elongation of the PHF-treated sample is primarily related to the unique transformation behavior of the strengthening phases in the alloy. During the pre-aging treatment of the PHF process, a large number of GP zones will be formed in the alloy, and in the subsequent hot forming at 200 °C, the precursor GP zones are difficult to completely transform into β″, β′ and β phases, while some unstable GP zones may even dissolve, both of which lead to a reduction in the content of the strengthening phases and subsequently a decrease in alloy strength. Since the GP zones and β″ phases present in the alloy are usually smaller in size than other precipitated phases, and larger precipitates are more likely to act as fracture initiation sites during tensile testing [6,29], the incomplete aging in PHF is noticeably beneficial to improving the alloy elongation. Moreover, as discussed above, although the average size of the second-phase particles in the two samples is almost the same, the size distribution in the PHF-treated sample is more concentrated, and the proportion of small particles is larger. The fine and evenly distributed second phase contributes to the enhancement of the tensile plasticity of the alloy.

The product of strength and elongation is an important index to describe the comprehensive mechanical properties of the structural materials [30]. For the two samples studied in this experiment, the corresponding values are 2.39 GPa·% and 2.68 GPa·%, respectively. That is, the PHF can not only greatly shorten the processing time and improve the production efficiency but is also expected to enhance the comprehensive mechanical properties of the material. Compared with the traditional process, the product of strength and elongation of the sample treated by PHF under the given processing conditions was increased by about 12.1%. It is clear that the improvement effect is expected to be further improved by optimizing the parameters of pre-aging and subsequent hot forming in the PHF process.

**Figure 9:**
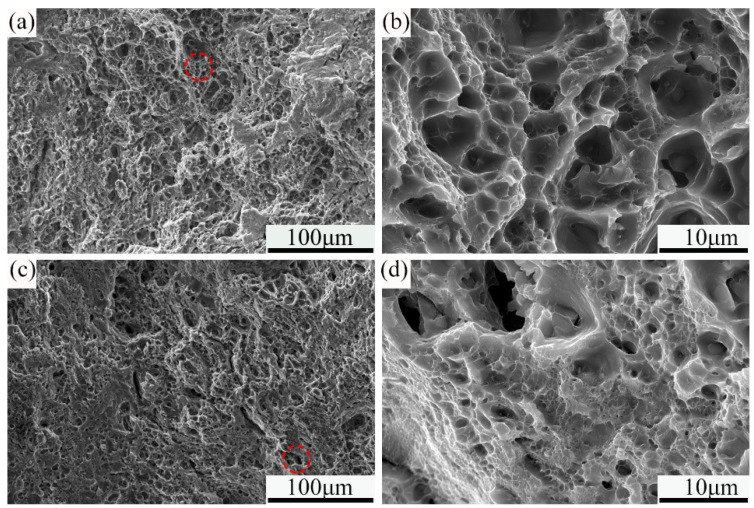
Typical tensile fracture morphologies of the samples treated with different processes. (**a**,**b**) Traditional; (**c**,**d**) PHF.

Figure 9 shows the typical tensile fracture morphologies of the samples treated with different processes. As can be seen from the figure, both the two samples exhibit ductile fracture characteristics. For the one treated by traditional process, large dimples appear on the tensile fracture, and second-phase particles can be observed at the bottom of some dimples, as shown in Figure 9a,b. These particles may act as initiation sites for fracture during tensile testing. Additionally, tear ridges with blunt patterns can also be seen on the fracture surface, suggesting some brittle fracture features. As illustrated in Figure 9c,d, the dimples in the PHF-treated sample were significantly smaller. The higher the density and smaller the size of the dimple in the tensile fracture, the better the plasticity of the alloy, which is consistent with the above tensile test results.

## 4. Conclusions

The microstructure and mechanical properties of the 6082-0.2Zr-0.3Er alloy treated with different processes were characterized and measured. Based on the experimental results and relevant theoretical analysis, the feasibility and corresponding action mechanisms of PHF applied to this aluminum alloy were discussed. The main conclusions can be summarized as follows:

1. Different from the traditional process, the grains in PHF-treated alloy show a certain directional arrangement, and the second phase precipitated on the grain boundary is also the same. In addition, the texture coefficients of (220) and (200) were enhanced after PHF treatment.
2. Although the average size of the second phase is almost the same in the two samples, the size distribution is more concentrated in the PHF-treated sample, with a higher proportion of finer particles. The fine and evenly distributed second phase is beneficial for enhancing the mechanical properties of the alloy, particularly the plasticity.
3. Compared with the traditional process, the product of strength and elongation of the sample treated by the PHF process increased by about 12.1%. PHF process can not only greatly shorten the processing time and improve the production efficiency but also is expected to improve the comprehensive mechanical properties of this 6082-0.2Zr-0.3Er alloy.
4. It has been proved that the PHF process is helpful in improving the comprehensive mechanical properties of 6082-0.2Zr-0.3Er alloy. However, the strength of the alloy decreased slightly in this study, so the pre-aging and hot-forming parameters are expected to be optimized to further improve the mechanical properties.

## Figures and Tables

**Table 1:** Chemical composition of the alloy.

Composition	Mg	Si	Mn	Cu	Zn	Cr	Fe	Ti	Zr	Er	Al
Content (wt.%)	0.8	1.1	0.8	0.1	0.2	0.25	0.5	0.1	0.2	0.3	Bal.

**Table 2:** Different processing parameters.

Process	Heat Treatment		Compression	
	Solution	Aging	Temperature	Deformation
Traditional	540 °C 1 h	175 °C 8 h	Room temperature	10%
PHF	540 °C 1 h	120 °C 4 h	200 °C	10%

**Table 3:** Texture coefficients (%) of the samples treated with different processes.

Sample	Tc (111)	Tc (200)	Tc (220)	Tc (311)
Traditional	4.7	9.2	72.5	13.6
PHF	1.4	12.3	74.8	11.5

**Table 4:** EDS point analyzing results of different locations shown in Figure 7a (at%).

Point	Al	Si	Mg	Mn	Cr	Fe	Zr	Er
1	76.42	10.38	1.18	8.27	2.19	1.41	-	0.15
2	87.17	6.73	1.19	0.15	0.12	0.04	-	4.6
3	96.7	0.52	1.94	0.44	0.22	0.13	-	0.05

**Table 5:** The yield strength, UTS, and elongation were obtained from the engineering stress–strain curves.

Sample	Yield Strength (MPa)	Tensile Strength (MPa)	Elongation (%)
Traditional	309	319	7.5
PHF	280	295	9.1

## Data Availability

We sincerely apologize that the data is unavailable due to our failure to create new data.
